# Editorial: Pulmonary hypertension in the modern era: Science and clinical practice, Volume II

**DOI:** 10.3389/fmed.2023.1136157

**Published:** 2023-02-06

**Authors:** Harm J. Bogaard, Elena A. Goncharova, Vinicio A. de Jesus Perez

**Affiliations:** ^1^Department of Pulmonary Medicine, Amsterdam University Medical Center, Amsterdam, Netherlands; ^2^Division of Pulmonary, Critical Care and Sleep Medicine, Lung Center, Department of Internal Medicine, Davis School of Medicine, University of California, Davis, Davis, CA, United States; ^3^Division of Pulmonary, Allergy and Critical Care Medicine, Stanford University Medical Center, Stanford, CA, United States

**Keywords:** pulmonary hypertension, right ventricle, microbiome, COVID-19, senescence, metabolism, inflammation

In 2021, this journal launched a Research Topic dedicated to translational science and clinical practice in pulmonary hypertension. Volume I of this topic was highly successful, with many valuable contributions from scientists across the globe. Because not all submitted papers could be accommodated in this first Volume, Frontiers in Medicine released Volume II of this topic on Pulmonary Hypertension.

Pulmonary hypertension is a devastating condition of high pulmonary pressures and right heart failure that, if untreated, leads to premature death. Research into disease mechanisms and optimal treatment remain of highest importance. In this volume, researchers from China, the United States and Europe provide important new insights into the diagnosis and management of pulmonary hypertension. First, in a study from Kunming, China, the microbiome in broncho-alveolar lavage fluid is compared between normal controls and pediatric patients with pulmonary arterial hypertension (PAH) associated with congenital left to right shunts (Ma et al.). The authors show an altered spectrum of microbes in pediatric PAH patients that is strongly correlated to a disturbed metabolomic profile. They speculate that metabolomic biomarkers may inform on clinical diagnosis, treatment, and prognosis of pediatric PAH patients. This volume also contains two valuable contributions on the diagnosis of chronic thromboembolic hypertension (CTEPH). Clinical scientists from Stanford University, Palo Alto, USA, conducted a survey to assess agreement amongst clinicians for initial therapy choice in patients with pulmonary hypertension in the context of chronic lung disease, or group 3 pulmonary hypertension (Thomas et al.). Although management guidelines discourage the routine use of PAH-targeted therapies in these patients, over 90% of respondents agreed that they would treat cases with severe group 3 pulmonary hypertension with some form of PAH-targeted therapy. For mild pulmonary hypertension and mild lung disease cases, <50% of respondents chose to start PAH-specific therapy. There was overall poor agreement between respondents in the choice to use mono-, double, or triple combination therapy with PAH-specific agents. This study shows wide practice variation and a strong need for further studies in this field. Le Pennec et al. from France address the current lack of consensus on the use of specific criteria for the interpretation of Ventilation/Perfusion (V/Q) lung scintigraphy to screen for CTEPH. In a group of 226 patients with newly diagnosed pulmonary hypertension, of whom one quarter were ultimately diagnosed with CTEPH, the optimal diagnostic cut-off for interpretation was 2.5 segmental mismatched perfusion defects. An interpretation based on perfusion defects only provided similar sensitivity but lower specificity. As scintigraphy retained its central position in the diagnosis of CTEPH in the new ERS/ESC pulmonary hypertension guidelines, this study provides valuable additive guidance for a proper diagnosis of CTEPH. Shahin et al. from Sheffield, UK, described the use of cardiac magnetic resonance the assess prognosis and predict the response to pulmonary endarterectomy (PEA) in CTEPH. While right ventricular ejection fraction predicted outcome in patients not undergoing PEA, measures of left atrial volume index were associated with outcome after PEA surgery. This study highlights the prognostic value of imaging cardiac structure and function in CTEPH and the importance of considering left heart disease in patients considered for PEA.

The COVID-19 pandemic has brought forth a new disease entity whose long-term impact on public health continue to evolve as many survivors are now suffering from Post-Acute Sequelae of SARS-CoV-2 (PASC), a condition in which individuals experience a wide range of physical and mental symptoms after their initial infection. The manuscript by Oliveira et al. focuses on the cardiopulmonary sequelae of COVID-19 infection which include right ventricular dysfunction, pulmonary hypertension, thrombosis, and lung fibrosis. Acknowledging the many knowledge gaps pertaining to these topics, the authors provide a list of research priorities to direct attention to the most urgent questions to be addressed in an effort to anticipate the health care needs that these patients will require as the pandemic continues to evolve over the next few years.

Moving from clinical to the basic sciences, the study by Kyi et al. included in the collection tackles the contribution of cellular senescence in triggering vascular cell accumulation in the distal pulmonary arterioles of PAH patients. In a series of elegant experiments, the investigators demonstrate that accumulation of the senescence marker p16^INK4a^ in pulmonary endothelial cells drives the production of endothelial-derived growth factors (e.g., PDGF) that promote the expansion of smooth muscle cells in the medial layer, a phenomenon that can be attenuated in *p16* ^fl/*fl*^*-Cdh5(PAC)-Cre* ^ERT2^ (*p16* ^*iΔEC*^) mice after tamoxifen induction. These studies provide compelling evidence that targeting senescence could be a viable therapeutic strategy in the management of PAH. Further, the study by Lin et al. takes a bioinformatics-based strategy to interrogate four publicly available datasets in an effort to identify prospective biomarkers and therapeutic targets in PAH. Among the genes identified in their robust analysis, TXNRD1 was selected for validation given its predictive performance and correlation with several hemodynamic parameters of PAH. Beyond the identification of TXNRD1, the most important aspect of the work is the demonstration that application of bioinformatics can accelerate the discovery of new mechanisms and targets using accumulating datasets being generated from Omics studies.

Continuing to uncover molecular mechanisms driving hyper-proliferation of pulmonary vascular cells in PAH, recent study by Jiang et al. investigated the role of lipogenesis in increased proliferation and apoptosis resistance of PAH PA smooth muscle cells. The authors found that, in contrast to healthy subjects, distal PA smooth muscle cells from PAH patients' lungs have enhanced lipogenesis, which is supported by up-regulation of key fatty acid synthesis enzymes ATP-citrate lyase, acetyl-CoA carboxylase, and fatty acid synthase. Importantly, the authors demonstrate that active lipogenesis is required for increased proliferation and survival of PA smooth muscle cells in PAH and provide strong evidence that there is a mechanistic link between glycolysis, lipogenesis, and the proliferation of human PAH PA smooth muscle cells, which is regulated by SIRT7/JNK-Akt axis. This study offers strong molecular basis for further investigations to determine the potential attractiveness of targeting abnormal lipogenesis to reduce pulmonary vascular remodeling and PAH.

Inflammation plays important role in PAH pathogenesis and supports multiple molecular and metabolic abnormalities involved in the initiation and progression of this deadly disease. The review article from Foley et al. discusses the role of inflammasome activation in the pathogenesis of PAH. Inflammasomes are multi-protein complexes that initiate and amplify immune responses induced by infectious or sterile inflammatory stimuli. Aberrant activation of inflammasomes has been implicated in various pulmonary and cardiovascular diseases. This timely and important review provides detailed overview of our current knowledge about inflammasome activation in immune and resident pulmonary vascular cells as it relates to the pathogenesis of PAH. The authors also provide in depth discussion of potential drug development to inhibit inflammasomes and their downstream effectors to treat PAH. Further emphasizing important role of inflammation and translational significance of transforming growth factor-β (TGF-β) superfamily signaling in dysregulated vascular cell proliferation in PAH, Andre et al. provide comprehensive review of the TGF-β superfamily mechanisms in PAH pathogenesis and expertly summarize the interaction of TGF-β superfamily signaling with the inflammation and mechanobiological forces. The authors also discuss emerging therapeutic strategies to restore SMAD signaling and reverse pulmonary vascular remodeling and overall PAH.

The right ventricle (RV) dysfunction is one of the most important determinants of survival in patients with pulmonary hypertension, but the age-related RV changes either in health or disease are not well-understood. To note, aging is associated with alterations in pulmonary vasculature and RV hemodynamics, and there is higher prevalence of pulmonary hypertension in the elderly. Using male Sprague-Dawley rats as a model, Sharifi Kia et al. report their pilot findings demonstrating effects of healthy aging on the structure, function, and biomechanical properties of RV. Specifically, the authors provide strong evidence suggesting that healthy aging could promote RV remodeling *via* increased peak pressures, cardiomyocyte loss, fibrosis, fiber reorientation, and altered mechanical properties. These findings improve our understanding of age-related changes in the RV fitness, which may play important role in response to disease, and call for further investigations to determine how age contributes to the disease progression in patients with pulmonary hypertension.

## Author contributions

All authors listed have made a substantial, direct, and intellectual contribution to the work and approved it for publication.

